# Changes in Physical Fitness during the COVID-19 Pandemic in German Children

**DOI:** 10.3390/ijerph19159504

**Published:** 2022-08-02

**Authors:** Tanja Eberhardt, Klaus Bös, Claudia Niessner

**Affiliations:** Institute of Sports and Sports Science, Karlsruhe Institute of Technology, 76131 Karlsruhe, Germany; klaus.boes@kit.edu (K.B.); claudia.niessner@kit.edu (C.N.)

**Keywords:** motor performance, motor development, youth, effects, influences, cross-sectional study

## Abstract

With the beginning of the COVID-19 pandemic in December 2019, each country has developed strategies to try to control the virus. The restrictions and subsequent consequences also limited the possibilities and structures for being physically active. Therefore, the aim of this study was to examine changes in physical fitness in a cohort that was investigated over an extended period. Physical fitness testing was conducted with the IPPTP-R in a primary school from a small rural community annually since 2012. Mean values of test items were calculated for each cohort. We conducted an ANCOVA to examine the differences between cohorts PreCOVID and 2020 as the first year of the COVID-19 pandemic, and between PreCOVID and 2021 as the second year of the COVID-19 pandemic. Overall, no evidence for a negative effect of the COVID-19 pandemic on physical fitness in children between the ages of 7 and 9 years was found. In strength tests, performances increased when comparing the PreCOVID cohort and COVID-19 cohorts (Push-Ups: *p* < 0.001, η_p_^2^ = 0.032; *p* = 0.017, η_p_^2^ = 0.006). No evidence for a change was found for endurance (6-min Run: *p* = 0.341, η_p_^2^ = 0.001; *p* = 0.267, η_p_^2^ = 0.001. The rural community maintained physical fitness despite restrictions and limitations through the environmental circumstances. Considering this, it is a positive example of how adequate long-term efforts promoting physical fitness make an impact and an active friendly environment helps to overcome COVID-19 pandemics limiting the structures for being physically active.

## 1. Introduction

The importance of physical fitness for the healthy development of children and the positive influence for a lifelong active lifestyle are well-known and documented [1,2]. The level of physical fitness predicts an individual’s level of engaging in physical activity through given opportunities and limited capacities [3,4]. Physical fitness is the basis on which movement patterns are developed to be able to be physically active and, on the other hand, has a positive impact on psychosocial factors [5,6,7,8].

Nevertheless, the levels of physical activity and physical fitness in youth have declined over the last decades, along with other variables influencing an active lifestyle. Since the beginning of the twenty-first century, physical fitness has been stagnating at a low level worldwide. Overall, children are less fit than those of former generations [9,10,11,12]. Accordingly, the majority of children and adolescents do not meet the recommendations of the World Health Organization for daily physical activity [13,14]. Sedentary behavior of children and adolescents has increased and screen-time exceeds recommendations [15,16]. As a consequence, the prevalence of obesity and overweight has steadily increased in past years, especially in younger children [17].

With the beginning of the COVID-19 pandemic in December 2019, each country has developed a strategy to try to control the virus. In Germany, the first officially registered case of COVID-19 appeared at the end of January 2020. Nationwide school closures and contact restrictions were implemented for the first time in March 2020, and again in December 2020 until March 2021 [18]. These restrictions also affected sports clubs, fitness centers, and the cancellation of all sports in schools, i.e., physical education lessons, extracurricular sports groups, or being active during breaks in the schoolyard. The COVID-19 pandemic and the subsequent consequences therefore not only limited social life, but also the possibilities and structures for being physically active.

There are studies that have examined the influence of the COVID-19 pandemic on physical activity [19,20]. A meta-analysis revealed a slightly negative global change in physical activity for children and adolescents [19]. In Germany, the differentiated analysis of data from the Motorik–Modul (MoMo) study showed an increase of daily physical activity, such as playing outside or unstructured activities, during the first lockdown, but children could not maintain this level during the second lockdown [21]. In contrast, the time spent in organized sports and overall physical activity decreased over the study period [20,21,22].

There are also some studies that examined the influence of the COVID-19 pandemic and associated restrictions not only on physical activity, but also on the physical fitness construct [23,24,25,26]. Despite different measurement methods and study participants, the studies all reported a declining trend for endurance [23,24,27]. There appears to be less and inconclusive evidence for decreasing strength [24,25]. However, most of the studies have single measurement points before, during, or after the COVID-19 pandemic, but there is a lack of long-term monitoring. In our study, we conducted physical fitness testing in the same cohort over a period of eight years, plus 2020 and 2021, years in which the COVID-19 pandemic occurred. Therefore, these cohorts, which constitute the specific study population, provide the opportunity to draw conclusions based on a strong foundation of physical fitness data.

The aim of the study was to examine effects of the COVID-19 pandemic and changes in the different dimensions of physical fitness in a cohort that was investigated over an extended period.

## 2. Materials and Methods

This study used a cross-sectional cohort design with a population-based ad hoc sample. Overall, ten cohorts were followed yearly from 2012 until 2021. In the following, cohort always refers to the age group of 7–9-year-olds in the respective testing year. The International Physical Performance Test Profile—revised (IPPTP-R) was used to test the physical fitness in in the German federal state of Baden-Württemberg [28]. All data presented in this paper were from children from a small rural community with fewer than 5000 inhabitants located in the northeast of Baden-Württemberg that participated in the test procedure over the entire period of ten years.

### 2.1. Physical Fitness

The IPPTP-R is an effective and validated physical fitness assessment tool developed to be conducted in practical settings [28]. It is based on the approach of Bös and Mechling [29] and the German Motor-Test 6–18 [30]. It contains eight test items representing the five main dimensions of physical fitness endurance, strength, speed, coordination, and flexibility. Additionally, constitutional data including height, weight, and BMI were collected, and children’s age and sex, as well as test date and other characteristics of data collection were recorded. Table 1 shows the different test items. The detailed and precise description of the test items can be found in the existing manuals [28,30].

### 2.2. Data Collection

The primary school in the community reported on here conducted the testing annually in October, except in 2020, when testing was limited due to the COVID-19 lockdown. Therefore, the 2020 tests were conducted in December. The teachers and volunteers were trained as multipliers using manuals, test material, and additional support and to execute the test tools. On a testing day, each child was tested in the school, sorted according to class. Parents provided informed consent forms through the primary school that conducted the testing. With informed consent, the test results were entered into the evaluation software and any personalized raw data on children’s physical fitness were pseudonymized initially and checked for quality. The data set regarding this community was retrieved from the total data set using postal code as variable of allocation. The extracted data were then analyzed in a separate dataset.

### 2.3. Sample Description

As mentioned above, all data were from one community in the German state of Baden-Württemberg, which participated over the entire study period. Overall, 999 primary school children between the ages of 7 to 9 years (MV ± SD: age: 7.98 ± 0.82; weight: 29.0 ± 6.9 kg; height: 132.8 ± 7.5 cm) were included in the analysis. Among them, 55.6% (*n* = 555) were boys and 44.4% (*n* = 444) were girls. In the analysis, cohorts were compared to examine the effects and consequences of the COVID-19 pandemic on physical fitness levels. The different cohorts from the period between 2012 and 2019 were combined and considered representative of the physical fitness of children in the community before COVID-19. This cohort, called PreCOVID, comprised 801 children (MV ± SD: age: 7.97 ± 0.82; weight: 28.8 ± 7.0 kg; height: 132.7 ± 7.4 cm). The cohort from 2020, the first year of the COVID-19 pandemic, called COVID1, included 91 children in the analysis (MV ± SD: age: 7.93 ± 0.87; weight: 28.9 ± 5.7 kg; height: 132.7 ± 7.8 cm). The cohort from 2021 (COVID2) included 107 children (MV ± SD: age: 8.08 ± 0.77; weight: 30.2 ± 7.1 kg; height: 133.4 ± 8.1 cm). The exact number of children according to cohort and gender is shown in Table 2.

### 2.4. Statistical Analysis

Statistical analyses were performed using IBM SPSS Statistics 28 (IBM Corporation, Armonk, NY, USA). To obtain an overview of the distribution within the sample, frequency analyses and cross tables were conducted.

Descriptive statistics with mean values and 95% CI were calculated for each test item and cohort overall and separately for boys and girls to reflect the entire measurement period. Missing data were not interpolated. The analysis was controlled for age and BMI. We conducted a univariate analysis of covariance (ANCOVA) to examine the differences between the cohorts PreCOVID and COVID1, and between PreCOVID and COVID2 adjusted for gender. The PreCOVID cohort value was formed using the mean value of individual cohorts from 2012–2019. The level of significance was set at *p* < 0.05. Effects were assessed with partial eta squared (η_p_^2^). Pairwise comparisons with Bonferroni correction were performed to determine differences between the cohorts.

## 3. Results

Overall, 999 primary school children aged 7 to 9 years from a small rural community in the German state of Baden-Württemberg were included in the study (PreCOVID *n* = 801; COVID1: *n* = 91; COVID2: *n* = 107). There were no significant differences for age and BMI between the ten cohorts, but gender-specific differences in mean values in the test items.

**Figure 1:**
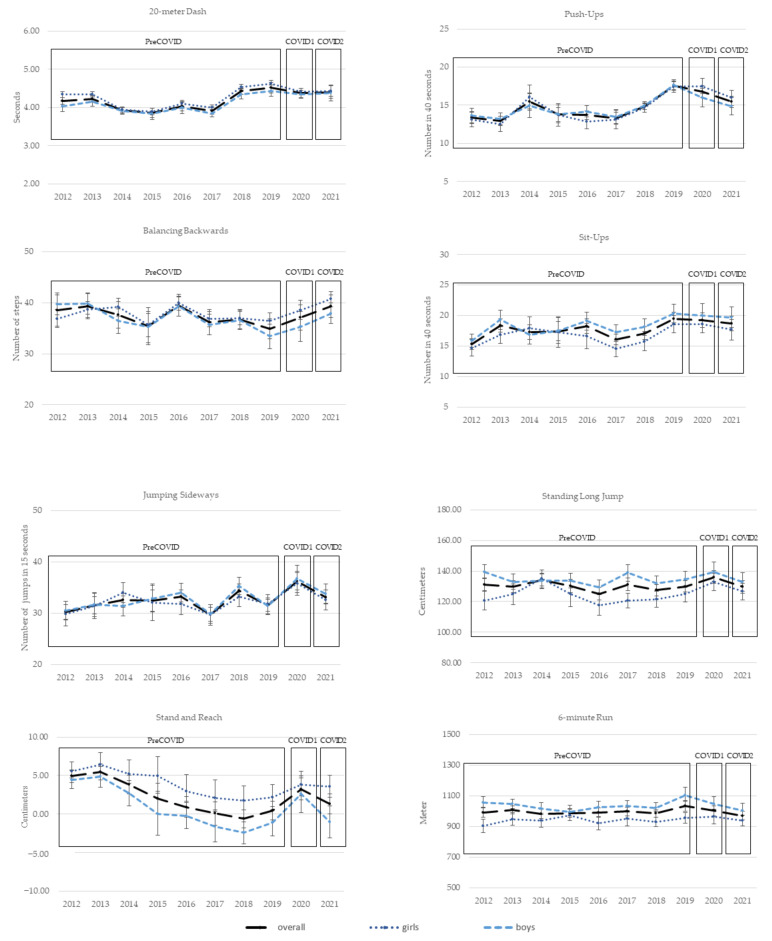
Trends for test items over each year of measurement overall, and for boys and girls separately.

Figure 1 shows the trends in test items for all measurement years overall and separately by gender (see Table A1).

There was a linear, consistent level of performance for the 20 m dash through 2017 in boys and girls. In 2018, an increase was observed for either gender, and this level of speed remained stable until the last measurement in 2021. The ANCOVA for the test item 20 m dash revealed that children in the cohort COVID1 were 0.20 s slower than in the PreCOVID cohort (F(1,859) = 15.89; *p* < 0.001; η_p_^2^ = 0.018). The comparison with cohort COVID2 showed a significant difference of 0.23 s (F(1,861) = 18.69; *p* < 0.001; η_p_^2^ = 0.021). The influence of the covariate gender was significant for both ANCOVAs (*p* < 0.001; *p* < 0.001).

The analysis of the test item balancing backwards showed an opposite gender-specific trend until 2015, followed by a peak in 2016 for both boys and girls. This increase stopped abruptly and tended to remain stable until 2019. No significant difference was found in the ANCOVA, with 0.22 steps between cohort PreCOVID and COVID1 (F(1,874) = 0.06; *p* = 0.813; η_p_^2^ = 0.000). However, children in cohort COVID2 performed 2.03 steps better than cohort PreCOVID (F(1,893) = 5.67; *p* = 0.017; η_p_^2^ = 0.006). The covariate gender had no significant influence on the analysis (*p* = 0.232; *p =* 0.215).

The mean values for jumping sideways revealed no trend. There were ups and downs through all cohorts, with a peak in 2020 and a minimum of performance in 2012 and 2017. There were inverse performance levels for boys and girls for the cohorts 2014 through 2016. Analyzing the differences for the test item jumping sideways showed that there was a difference of 4.32 fewer steps in PreCOVID compared with COVID1 (F(1,874) = 27.05; *p* < 0.001; η_p_^2^ = 0.030). There was no significant difference found between PreCOVID and COVID2, with 1.24 fewer steps measured for PreCOVID (F(1,892) = 2.71; *p* = 0.100; η_p_^2^ = 0.003). The influence of the covariate gender was not significant (*p* = 0.423; *p* = 0.353).

A steadily declining trend was found for the test item stand and reach, with its minimum in 2018. The level of flexibility subsequently increased. This development was found for boys and girls equally, but with clear differences in the measured values. The ANCOVA revealed 0.88 cm more in COVID1 than in cohort PreCOVID, but the difference was not significant (F(1,871) = 1.44; *p* = 0.230; η_p_^2^ = 0.002). PreCOVID had 1.00 cm less for stand and reach than COVID2, but this difference was also not significant (F(1,888) = 2.16; *p* = 0.142; η_p_^2^ = 0.002). The covariate was statistically significant in both cohort comparisons (*p* < 0.001; *p* < 0.001).

Push-up performance was consistent over the cohorts before increasing in 2017 and peaking in 2019. A significant difference was found in COVID1 with 2.29 more performed push-ups compared with PreCOVID (F(1,875) = 28.63; *p* < 0.001; η_p_^2^ = 0.032). There was a significant positive difference between PreCOVID and COVID2 of 0.94 (F(1,892) = 5.69; *p* = 0.017; η_p_^2^ = 0.006). The covariate gender had no significant influence on either ANCOVA (*p* = 0.855; *p* = 0.924).

The test item sit-ups improved consistently, but showed an apparent reversal in gender-specific performance for 2014 and the highest levels up to 2019. Analyzing cohort differences with the ANCOVA, the performance differed significantly with 1.95 more sit-ups in COVID1 than in PreCOVID (F(1,863) = 9.87; *p* = 0.002; η_p_^2^ = 0.011). In addition, with 1.36 more sit-ups, COVID2 was significantly better than PreCOVID (F(1,881) = 5.50; *p* = 0.019; η_p_^2^ = 0.006). The covariate gender had a significant influence on the differences (*p* < 0.001; *p* < 0.001).

For standing long jump, initial measurements already showed significant differences between boys and girls. This difference was found for all cohorts with no apparent trend. However, the analysis revealed that children in COVID1 jumped 7.24 cm farther than children in PreCOVID (F(1,873) = 11.13; *p* < 0.001; η_p_^2^ = 0.013). However, the comparison between PreCOVID and COVID2 showed no significant difference, with 0.56 cm more for COVID2 (F(1,890) = 0.08; *p* = 0.782; η_p_^2^ = 0.000). The covariate gender was statistically significant for both cohort comparisons (*p* < 0.001; *p* < 0.001).

The mean values for 6 min run in the 2012 cohort differed significantly for boys and girls. Performance differed by gender over the measurement period, but the development of the overall sample revealed no trend. No significant differences were found between COVID1 and PreCOVID for the 6 min run, with COVID1 running only 15.31 m more than PreCOVID (F(1,857) = 0.91; *p* = 0.341; η_p_^2^ = 0.001). The children in PreCOVID ran 16.94 m less than COVID2, but these differences were also not significant (F(1,866) = 1.23; *p* = 0.267; η_p_^2^ = 0.001). The covariate gender had a significant influence on the measured differences (*p* < 0.001; *p* < 0.001). See Table A2 and Table A3 for the adjusted mean values of the ANCOVA in each test item and cohort.

## 4. Discussion

The aim of the study was to examine COVID-19 effects on the physical fitness of primary school children in a rural community in Baden-Württemberg with fewer than 5000 inhabitants. We conducted measurements over a long-term period and therefore have strong evidence for the overall levels and changes of physical fitness in the sample.

### 4.1. Summary and Evaluation of the Results

For the test item 20 m dash, the statistically significant differences had no practical relevance. Speed levels remained stable in the COVID-19 cohorts. Comparing the results for balancing backwards, we see that the previously constant performance increased in the measurement for COVID2, but remained the same in COVID1. In addition, representing the dimension coordination, jumping sideways also had a peak in COVID1, but the same stable level before and after. Thus, no evidence for an effect was found. Pombo et al. [31] also reported no inferior results for jumping sideways in 6–9-year-old Portuguese children tested before and after the COVID-19 lockdown. However, from December 2019 to September 2020, there was an overall general trend of shifting to a lower quartile [31]. An increase in children’s performance categorized as “very low” in the 20 m shuttle run was also observed by Basterfield et al. for participants in a primary school in England [25]. Flexibility performance in the stand-and-reach test did not differ significantly between the three cohorts. The rising trend of recent years was not stopped by the pandemic. In contrast, there was a negative effect in the study in England, which measured a decrease of 1.8 cm between October 2019 and November 2020 for 8–10-year-old children [25].

The number of performed push-ups was significantly higher in both COVID cohorts compared to the overall 2012–2019 pre-pandemic cohort, which was also reflected in the results of the sit-ups test. There was no evidence that upper body strength levels were influenced negatively by COVID-19 restrictions and consequences. While Wahl-Alexander and Camic [27] found a decrease of 35.6% for push-ups and 19.4% for sit-ups in children with a mean age of 9.6 years between summer 2019 and 2020, other results are consistent with our findings. The same was found for leg strength, which was measured using the standing long jump test. Performance levels increased significantly in the COVID1 cohort, but then remained stable again compared to the PreCOVID cohort, and showed no evidence of negative effects. Basterfield et al. [25] and Wessely et al. [32] also reported a performance increase for standing long jump. This suggests that strength is more resilient to negative effects of COVID-19 than other dimensions of physical fitness [25]. However, Chambonnière et al. [24] measured the standing long jump performance of 3rd and 4th graders in France for the period between 2020 and 2021 and found a decrease of 34.7 cm. This is consistent with another study examining the same age between 2019 and 2020 [31]. 

The analysis for endurance revealed no significant difference between the cohorts and no effect of the COVID-19 pandemic. The performance levels for the 6 min run were stable across the measured cohorts. Other studies that analyzed endurance found different effects [23,25,27,31]. Jarnig et al. [23], who also implemented the 6 min run, reported a decrease of 102 m in children aged 7-to-10 years old between September 2019 and September 2020 [23]. Two other studies performed the 20 m shuttle run to measure effects for endurance and reported 2.39 [24] and 3 [25] fewer shuttles in their second measurement point.

### 4.2. Explanation Approaches

Overall, our results show no evidence for a negative effect of the COVID-19 pandemic on physical fitness in children between the ages of 7 and 9 years, but changes varied for the different test items and dimensions. Especially, performances of the COVID cohorts in test items for strength increased. It seems that alternative options of exercising physical fitness like online and indoor workouts mitigate some effects of COVID-19 pandemic. However, due to the restrictions and closures of organized forms in sports clubs or schools, we conclude that dimensions where high intensities and stimuli are needed could not benefit. This could suggest the relevant role of physical activity with peers and within an institution to maintain a global and comprehensive development in all dimensions of physical fitness.

When classifying the data into gender and age-specific percentiles of a nationwide reference sample, the children in this specific community represent a very high level of physical fitness above the average [33,34]. The community has various specific initiatives and commitments to promote physical activity. For example, the primary school curriculum emphasizes the importance of physical fitness and appropriate promotion is determined in the preamble. The community is also a part of the project “Bewegte Kommune-Kinder” which aims to enable a sufficient and adequate development of physical fitness for all children in the community. It seems that children who had higher levels of physical fitness before COVID-19 are more resilient with regard to restrictions and limitations affecting physical activity. Similary, Jarnig et al. [23] reported that children who were members of sports clubs had better cardiorespiratory fitness measures at all time points. However, a higher level in the beginning leads to a higher level after the pandemic [23]. Adequate levels of physical fitness appear to increase resilience to limited physical activity due to external circumstances, such as the lockdowns during the COVID-19 pandemic.

Furthermore, there is evidence that total physical activity did not decline globally during the COVID-19 pandemic but that the form of being physically active changed [19,20,21,35]. While organized physical activity decreased, time spent in habitual physical activity and unstructured forms such as playing outside increased [20,22,35]. Schmidt et al. [22] found an increase from 75 min per day before the COVID-19 pandemic to 105 min per day playing outside during lockdown in spring 2020 for 6-to-10-year-old children in Germany. Most notably, socioeconomic background and place of residence are influencing determinants of levels of physical activity and physical fitness [19,20,32,35]. We also analyzed physical activity changes in our sample and can confirm these findings. Children indicated that they spent less minutes for physical activity in sports clubs, while time for physical activity in leisure time increased during the COVID-19 pandemic [36].

Wessely et al. [32] reported decreasing results for measurements of physical fitness during the COVID-19 pandemic, whereby children with a high social burden showed lower performance levels. Children with low socioeconomic status also showed lower levels of physical activity, but the home and living environment had a particular influence [19,20]. In our study, we have no data on socioeconomic status of the study subjects, but we consider community structure data. The community has less than 5000 inhabitants and is located in the north-east of Baden-Württemberg in Germany. The environment is known as rural with access to green areas and playgrounds. For the children in this community, the environment might have provided easy opportunities to be physically active during the COVID-19 pandemic and thus possibly prevented a negative effect on physical fitness. For rural children, the impact of COVID-19 policies and restrictions was limited, but results may differ in urban children. Asked with whom they were physically active, more children in this community named their parents, when restrictions were issued [36].

### 4.3. Limitations

There are limitations regarding sample and selection bias since we investigated an ad hoc sample with a cross-sectional cohort design of children from one primary school in a rural community of the German federal state Baden-Württemberg. The sample is not representative and its results show a selection effect concerning a higher physical activity and fitness compared with the whole of Germany. However, the long study period and the number of cohort measurements before and during the COVID-19 pandemic ensure that children’s physical fitness is considered globally and not just a one-point statement based on a one-point measurement.

Moreover, in this study were some confounding factors, e.g., socio-economic status, educational level of parents, and level of testosterone, which may affect the results, but were not controlled. However, we can use some physical activity data to classify. For future investigations, the methodology should be improved and possible cofounding data collected.

### 4.4. Practical Implications

The results showed that this particular community, which has been testing and supplementing physical fitness promotion with additional projects for ten years, has an above-average level of physical fitness. The data thus suggest that a variety of long-term physical fitness programs really do help a lot when it comes to promoting an active and healthy lifestyle. The programs should be anchored sustainably in the community and target people’s behavior and the conditions. Because not every child had the same opportunities to be physically active [18,32], especially in times when restrictions and limitations influence regular and structured physical activity, policy makers, communities, and other relevant stakeholders must provide children with access to environments that are conducive to and supportive of physical activity. Parents should operate as role models for an active lifestyle. Further research needs to examine larger cohort data to determine generalizable effects of the COVID-19 pandemic on physical fitness in children. In addition, these cohorts need to be monitored for additional years to establish long-term effects and influences. Pooling data, for example with the MO|RE data repository, from many small samples tested with a uniform and standardized measurement tool helps provide a wide range of participants and increases the comparability of findings across studies [37].

## 5. Conclusions

In conclusion, this study examined effects of the COVID-19 pandemic and changes in various dimensions of physical fitness in a cohort investigated over a long-term period of ten years. We found no evidence for an overall negative effect, but results differed between test items and dimensions. The rural community presented in this study is well aware of the importance of physical fitness. Physical fitness was maintained despite restrictions and limitations through the environmental circumstances. Considering this, this sample is a positive example of maintaining physical fitness throughout the COVID-19 pandemic. Adequate interventions and long-term efforts make an impact, but should address each child.

## Figures and Tables

**Table 1:** Test items of the IPPTP-R.

Dimension	Test Item	Unit
Endurance	6 min Run	Meter
Strength	Standing Long Jump	Centimeters
	Sit-Ups	Number in 40 s
	Push-Ups	Number in 40 s
Speed	20 m Dash	Seconds
Coordination	Balancing Backwards	Number of steps
	Jumping Sideways	Number of jumps in 15 s
Flexibility	Stand and Reach	Centimeters

**Table 2:** Distribution of the sample.

Cohort	Year of Measurement	Boys (*n*)		Girls (*n*)		Overall (*n*)	
2012–2019 PreCOVID	2012	57	*n* = 460 (57.4%)	44	*n* = 341 (42.6%)	101	*n* = 801 (100%)
	2013	62		39		101	
	2014	56		43		99	
	2015	39		28		67	
	2016	61		33		94	
	2017	57		45		102	
	2018	64		49		113	
	2019	64		60		124	
2020 COVID1	2020	43	*n* = 43 (47.3%)	48	*n* = 48 (52.7%)	91	*n* = 91 (100%)
2021 COVID2	2021	52	*n* = 52 (48.6%)	55	*n* = 55 (51.4%)	107	*n* = 107 (100%)
	Overall	555 (55.6%)		444 (44.4%)		*n* = 999 (100%)	

## Data Availability

The datasets presented in this study will be publicly archived at: http://motor-researchdata.org/.

## Appendix A

**Table A1:** Mean values for each test item and cohort overall and separately for boys and girls.

Test Item	Cohort	Mean Value (95% CI)		
		Male	Female	Overall
20 m	2012	4.03 (3.94–4.11)	4.33 (4.19–4.48)	4.16 (4.08–4.24)
	2013	4.15 (4.07–4.24)	4.35 (4.22–4.47)	4.23 (4.15–4.30)
	2014	3.91 (3.83–4.00)	3.94 (3.85–4.02)	3.92 (3.86–3.98)
	2015	3.83 (3.71–3.96)	3.89 (3.75–4.04)	3.86 (3.77–4.00)
	2016	3.99 (3.88–4.10)	4.10 (3.94–4.26)	4.02 (3.93–4.12)
	2017	3.85 (3.75–3.94)	4.00 (3.90–4.09)	3.91 (3.85–3.98)
	2018	4.34 (4.26–4.41)	4.54 (4.43–4.65)	4.42 (4.36–4.49)
	2019	4.43 (4.30–4.57)	4.62 (4.47–4.76)	4.52 (4.42–4.62)
	2020	4.34 (4.21–4.47)	4.41 (4.30–4.52)	4.38 (4.29–4.46)
	2021	4.38 (4.20–4.55)	4.43 (4.22–4.63)	4.40 (4.27–4.53)
BalBw	2012	39.74 (37.60–41.87)	36.86 (33.85–39.88)	38.49 (36.71–40.26)
	2013	39.80 (37.80–41.81)	38.64 (36.10–41.18)	39.35 (37.80–40.90)
	2014	36.39 (33.96–38.82)	39.14 (36.56–41.72)	37.61 (35.85–39.37)
	2015	35.23 (32.16–38.30)	35.61 (31.99–39.22)	35.39 (33.11–37.67)
	2016	39.27 (37.38–41.16)	39.91 (37.78–42.04)	39.50 (38.09–40.91)
	2017	35.73 (33.68–37.78)	36.79 (34.33–39.24)	36.18 (34.63–37.73)
	2018	36.61 (34.71–38.51)	36.98 (35.15–38.81)	36.77 (35.45–38.09)
	2019	33.43 (31.04–35.82)	36.41 (34.42–38.39)	34.87 (33.30–36.43)
	2020	35.33 (32.42–38.24)	38.56 (36.08–41.05)	37.11 (35.23–39.00)
	2021	37.90 (35.88–39.93)	40.72 (38.51–42.94)	39.34 (37.84–40.84)
JumpSw	2012	30.48 (28.64–32.32)	29.90 (27.50–32.30)	30.23 (28.78–31.68)
	2013	31.62 (29.37–33.86)	31.45 (28.96–33.94)	31.55 (29.90–33.20)
	2014	31.35 (29.41–33.29)	34.01 (32.04–35.98)	32.53 (31.14–33.92)
	2015	32.78 (30.20–35.37)	32.09 (28.49–35.69)	32.49 (30.42–34.56)
	2016	33.95 (32.14–35.76)	31.80 (29.77–33.84)	33.18 (31.81–34.55)
	2017	29.84 (27.98–31.71)	29.67 (27.64–31.70)	29.77 (28.42–31.13)
	2018	35.29 (33.59–36.98)	33.17 (31.23–35.10)	34.38 (33.11–35.53)
	2019	31.33 (29.70–32.97)	31.77 (29.90–33.64)	31.54 (30.32–32.76)
	2020	36.68 (34.03–39.34)	35.83 (33.53–38.14)	36.23 (34.51–37.94)
	2021	33.77 (31.86–35.68)	32.56 (30.59–34.54)	33.15 (31.79–34.51)
St&R	2012	4.48 (3.34–5.62)	5.54 (4.32–6.76)	4.94 (4.11–5.77)
	2013	4.84 (3.49–6.20)	6.40 (4.85–7.95)	5.45 (4.43–6.47)
	2014	2.70 (1.05–4.35)	5.20 (3.39–7.02)	3.81 (2.58–5.03)
	2015	−0.02 (−2.73–2.69)	4.94 (2.41–7.47)	2.01 (0.06–3.96)
	2016	−0.21 (−1.89–1.48)	2.97 (0.82–5.12)	0.90 (−0.44–2.24)
	2017	−1.60 (−3.59–0.39)	2.09 (−0.22–4.40)	0.07 (−1.46–1.60)
	2018	−2.43 (−3.84–−1.02)	1.74 (−0.16–3.63)	−0.62 (−1.81–0.57)
	2019	−1.13 (−2.82–0.56)	2.17 (0.55–3.79)	0.47 (−0.73–1.66)
	2020	2.57 (0.17–4.97)	3.82 (2.08–5.57)	3.25 (1.82–4.68)
	2021	−0.99 (−3.06–1.08)	3.54 (2.04–5.04)	1.32 (−0.01–2.64)
PU	2012	13.61 (12.63–14.60)	13.14 (12.13–14.15)	13.41 (12.71–14.10)
	2013	13.18 (12.39–13.98)	12.54 (11.55–13.53)	12.93 (12.32–13.54)
	2014	14.96 (13.37–16.56)	16.09 (14.56–17.63)	15.46 (14.36–16.57)
	2015	13.85 (12.68–15.01)	13.71 (12.21–15.21)	13.79 (12.89–14.69)
	2016	14.19 (13.43–14.94)	12.85 (11.92–13.77)	13.71 (13.11–14.30)
	2017	13.47 12.50–14.45)	13.14 (11.93–14.34)	13.33 (12.58–14.07)
	2018	14.94 (14.40–15.48)	14.67 (14.07–15.27)	14.82(14.42–15.22)
	2019	17.65 (16.93–18.37)	17.45 (16.73–18.17)	17.55 (17.05–18.06)
	2020	15.98 (14.75–17.20)	17.49 (16.48–18.50)	16.78 (16.00–17.57)
	2021	14.88 (13.77–16.00)	15.96 (14.99–16.93)	15.44 (14.71–16.17)
SU	2012	15.88 (14.75–17.00)	14.70 (13.34–16.06)	15.36 (14.50–16.23)
	2013	19.32 (17.76–20.87)	16.84 (15.41–18.27)	18.36 (17.24–19.47)
	2014	16.87 (15.29–18.44)	17.90 (16.06–19.75)	17.32 (16.14–18.50)
	2015	17.54 (15.41–19.67)	17.26 (14.73–19.78)	17.42 (15.84–19.01)
	2016	19.09 (17.60–20.57)	16.65 (14.61–18.68)	18.23 (17.03–19.43)
	2017	17.25 (16.01–18.49)	14.55 (13.29–15.81)	16.09 (15.18–17.01)
	2018	18.11 (16.79–19.43)	15.78 (14.19–17.36)	17.10 (16.08–18.12)
	2019	20.32 (18.76–21.88)	18.59 (17.22–19.97)	19.48 (18.44–20.52)
	2020	20.00 (18.08–21.92)	18.60 (17.18–20.01)	19.25 (18.09–20.41)
	2021	19.71 (18.02–21.40)	17.70 (16.03–19.37)	18.69 (17.51–19.87)
SLJ	2012	139.54 (134.95–144.13)	120.59 (114.53–126.65)	131.29 (127.21–135.37)
	2013	132.90 (127.89–137.92)	124.92 (118.14–131.71)	129.79 (125.74–133.84)
	2014	133.58 (128.50–138.67)	134.84 (128.92–140.76)	134.13 (130.34–137.92)
	2015	133.90 (129.10–138.70)	125.00 (116.76–133.24)	130.26 (125.85–134.66)
	2016	129.37 (124.65–134.09)	117.67 (110.92–124.42)	125.17 (121.20–129.15)
	2017	138.72 (133.39–144.05)	120.53 (115.73–125.32)	131.22 (127.15–135.29)
	2018	131.91 (129.87–136.94)	121.63 (116.18–127.07)	127.50 (123.73–131.27)
	2019	134.44 (129.30–139.59)	125.14 (119.90–130.37)	129.98 (126.27–133.70)
	2020	139.41 (133.13–145.70)	133.04 (127.39–138.70)	135.98 (131.80–140.15)
	2021	132.75 (126.53–138.97)	126.57 (121.29–131.86)	129.60 (125.56–133.65)
6 min	2012	1056.79 (1019.57–1094.01)	903.60 (859.31–947.90)	990.92 (959.09–1022.75)
	2013	1044.85 (1010.55–1079.14)	948.78 (907.96–989.61)	1007.82 (980.29–1035.35)
	2014	1016.37 (975.45–1057.28)	936.19 (893.23–979.15)	980.54 (950.28–1010.80)
	2015	996.31 (953.16–1039.46)	974.18 (936.37–1011.99)	987.06 (957.98–1016.14)
	2016	1026.43 (988.75–1064.10)	919.73 (876.69–962.76)	988.97 (958.83–1019.11)
	2017	1032.39 (996.43–1068.34)	950.41 (905.34–995.49)	997.01 (968.11–1025.91)
	2018	1022.42 (990.24–1054.60)	931.18 (900.59–961.77)	983.82 (959.87–1007.77)
	2019	1103.38 (1052.19–1154.57)	955.44 (920.26–990.62)	1031.84 (998.10–1065.57)
	2020	1045.39 (996.26–1094.52)	963.89 (918.40–1009.38)	1001.86 (968.03–1035.70)
	2021	1004.31 (956.22–1052.41)	939.65 (904.93–974.37)	969.65 (940.40–998.90)

20-m Dash: 20 m; Balancing Backwards: BalBw; Jumping Sideways: JumpSw; Stand and Reach: St&R; Push-Ups: PU; Sit-Ups: SU; Standing Long Jump: SLJ; 6-min Run: 6 min.

**Table A2:** Adjusted mean values of the ANCOVA in each test item- PreCOVID vs. COVID1.

Test Item	PreCOVID Mean Value (95% CI)	COVID1 Mean Value (95% CI)	Pairwise Comparisons Δ Mean Value (SD)
20 m	4.16 (4.13–4.19)	4.36 (4.27–4.46)	Δ +0.20 (0.05) *p* < 0.001
BalBw	37.26 (36.68–37.84)	37.04 (35.28–38.80)	Δ −0.22 (0.95) *p* = 0.813
JumpSw	31.95 (31.43–32.47)	36.27 (34.72–37.81)	Δ +4.32 (0.83) *p* < 0.001
St&R	2.09 (1.63–2.55)	2.98 (1.61–4.34)	Δ +0.88 (0.74) *p* = 0.230
PU	14.49 (14.23–14.76)	16.78 (15.98–17.58)	Δ +2.29 (0.43) *p* < 0.001
SU	17.45 (17.06–17.84)	19.40 (18.25–20.56)	Δ +1.95 (0.62) *p* = 0.002
SLJ	129.80 (128.43–131.14)	137.02 (132.99–141.06)	Δ +7.24 (2.17) *p* = 0.001
6 min	996.33 (986.26–1006.40)	1011.64 (981.75–1041.53)	Δ +15.31 (16.08) *p* = 0.341

20-m Dash: 20 m; Balancing Backwards: BalBw; Jumping Sideways: JumpSw; Stand and Reach: St&R; Push-Ups: PU; Sit-Ups: SU; Standing Long Jump: SLJ; 6-min Run: 6 min.

**Table A3:** Adjusted mean values of the ANCOVA in each test item- PreCOVID vs. COVID2.

Test Item	PreCOVID Mean Value (95% CI)	COVID2 Mean Value (95% CI)	Pairwise Comparisons Δ Mean Value (SD)
20 m	4.19 (4.13–4.19)	4.39 (4.29–4.49)	Δ +0.23 (0.05) *p* < 0.001
BalBw	37.26 (36.69–37.83)	39.29 (37.72–40.86)	Δ +2.03 (0.85) *p* = 0.017
JumpSw	31.95 (31.44–32.46)	33.19 (31.80–34.57)	Δ +1.24 (0.75) *p* = 0.100
St&R	2.09 (1.64–2.55)	1.10 (−0.15–2.34)	Δ −1.00 (0.68) *p* = 0.142
PU	14.49 (14.23–14.76)	15.44 (14.71–16.17)	Δ +0.94 (0.40) *p* = 0.017
SU	17.45 (17.06–17.84)	18.81 (17.74–19.88)	Δ +1.36 (0.58) *p* = 0.019
SLJ	129.80 (128.44–131.17)	130.36 (126.64–134.08)	Δ +0.56 (2.02) *p* = 0.782
6 min	996.23 (986.25–1006.22)	979.29 (951.07–1007.51)	Δ −16.94 (15.26) *p* = 0.267

20-m Dash: 20 m; Balancing Backwards: BalBw; Jumping Sideways: JumpSw; Stand and Reach: St&R; Push-Ups: PU; Sit-Ups: SU; Standing Long Jump: SLJ; 6-min Run: 6 min.
